# Analysis of Carotid color ultrasonography and high sensitive C-reactive protein in patients with atherosclerotic cerebral infarction

**DOI:** 10.12669/pjms.324.9731

**Published:** 2016

**Authors:** Lei Zhao, Zhanyi Zhai, Wei Hou

**Affiliations:** 1 Lei Zhao, Ultrasonic Department, Central Hospital of Zhumadian, Zhumadian City, Henan Province, China; 2 Zhanyi Zhai, Department of Severe Respiratory Disease, Central Hospital of Zhumadian, Zhumadian City, Henan Province, China; 3 Wei Hou, Ultrasonic Department, Central Hospital of Zhumadian, Zhumadian City, Henan Province, China

**Keywords:** High-sensitivity-CRP(hs-CRP), Intima–media thickness (IMT), Carotid plaque, Atherosclerotic cerebral infarction, Neurological function

## Abstract

**Objectives:**

To detect the correlation between high-sensitivity-CRP, carotid plaque, neurological function and intima–media thickness, and help physicians in the diagnosis of atherosclerotic cerebral infarction.

**Methods:**

A total of 96 patients with the first onset of atherosclerotic cerebral infarction were included in the study from July 2013 to May 2015. The test of high-sensitivity-CRP, examination of carotid color ultrasonography and neurological function evaluation were carried out for all the participants.

**Results:**

Ninety-six patients were divided into carotid plaque group and non-plaque group according to the existence of a carotid plaque after carotid artery ultrasonography. The carotid plaque group was further subdivided into stable plaque and unstable plaque subgroups according to plaque characteristics. The age in two subgroups was significantly higher than the non-plaque group (p<0.05). The unstable plaque subgroup presented with the highest values in intima–media thickness and high-sensitivity-CRP level, followed by stable plaque subgroup and non-plaque group (p<0.05). With the nervous damage scale increase, the level of high-sensitivity-CRP increase significantly (p<0.05). In addition, there was significant correlation between NIHSS score and high-sensitivity-CRP in patients with atherosclerotic cerebral infarction (p<0.05).

**Conclusion:**

The level of high-sensitivity-CRP and intima–media thickness is closely associated with the development of carotid plaque, and high-sensitivity-CRP can be regarded as a high sensitive index in deciding the risk and prognosis of atherosclerotic cerebral infarction.

## INTRODUCTION

Stroke, with high morbidity, mortality and disability rates, is one of the leading causes of death in the world. Of all the stroke cases, about 30% are fatal and 70% of the survivors have hemiplegia, aphasia or other disabilities,^1^ which lead to not only a serious threat to human health, but also a heavy burden to social security system.^2^ Cerebral infarction is the most common type of stroke, which even accounts for up to 43-79% of the whole stroke patients in China.^3^ Atherosclerosis is considered as the most important pathological factor for cerebral infarction by physicians all over the world.

Many researchers have suggested that inflammation plays an important role in the pathophysiological process of atherosclerosis and ischemic brain injury,^4^ indicates it is a key driver of atherosclerotic plaque development.^5^ Among all the inflammatory biomarkers, C-reactive protein (CRP) is an important inflammatory biomarker which can independently predict future vascular events. However, the sensitivity of CPR is relatively lower in clinical examination, so high-sensitivity (hs)-CRP is regarded as the best marker for predicting these events.^6^

Lots of cerebrovascular accidents result from a hemodynamic or thromboembolic event produced from carotid plaque.^7^ The correlation between inflammation, atherosclerosis progression and cerebrovascular events have been confirmed by many authors.^8^,^9^ Examination of plaques can be performed using CT, MRI, digital subtraction angiography and carotid ultrasonography, in which carotid ultrasonography has many advantages and is widely used as a reliable technique to image carotid atherosclerosis, and it can provide better definition of arterial lumen, plaque morphology and thickness, and intima–media thickness of carotid artery.^10^ Moreover, intima–media thickness is regarded as an index for the prediction of atherosclerosis.

As both high-sensitivity (hs)-CRP and carotid ultrasonography plays an important role in the diagnosis of atherosclerotic cerebral infarction, we speculate that these indices including hs-CRP and intima–media thickness may be close correlated in the diagnosis of atherosclerotic cerebral infarction. A study on these correlations may be helpful for physicians in diagnosing the fatal disease. However, few studies in this regard have been published.

Therefore, a prospective study was carried out in our hospital, and the objectives of the study were to detect the correlation between high-sensitivity (hs)-CRP, carotid plaque, neurological function and intima–media thickness, analyze the characteristics of these examinations, and help physicians in diagnosis of atherosclerotic cerebral infarction.

## METHODS

### Patients

A total of 96 patients with the first onset of atherosclerotic cerebral infarction were included in the current study from July 2013 to May 2015. The diagnosis of atherosclerotic cerebral infarction was performed according to the diagnostic criteria of the Fourth National Academic Conference on Cerebrovascular Disease,^11^ and the diagnosis was verified with CT or MRI. Those patients with non-atherosclerotic cerebral infarctions, hemorrhagic stroke, blood diseases, malignant tumors, autoimmune diseases, inflammatory diseases and a history of ischemic cerebrovascular disease were excluded from the study.^4^ The disease course of atherosclerotic cerebral infarction was not more than three days in all the included patients. All participants gave their written informed consent. This study was approved by the Ethics Committee of our hospital.

### Procedures

The test of high-sensitivity (hs)-CRP, examination of carotid color ultrasonography and neurological function evaluation were carried out for all the participants.

The carotid color ultrasonography was used to evaluate atherosclerotic plaque morphology and measure intima–media thickness. Both vertical and transverse scanning along the lateral edge of the sternocleidomastoid muscle were performed while the participant lay in supine position, with his head turned to the opposite side. Carotid plaques were defined as focal echogenic thickenings with a minimal intimal plus medial thickness ≥1.2 mm.^12^ A plaque with a rough surface was defined as an unstable plaque, and the one with a strong echo and smooth surface defined as a stable plaque.^13^

The hs-CRP level of the included participants was detected using an ultrasensitive nephelometric method with automatic biochemistry. Neurological function of patients was assessed with the National Institutes of Health Stroke Scale (NIHSS).^14^

### Statistical Analysis

Statistical analysis was carried out using SPSS21.0 (SPSS Inc., Chicago, IL, USA). The comparison of measurement data including age, level of hs-CRP, NIHSS scores, intima-media thickness of common carotid artery was performed using t test between two groups. The difference of enumeration data was evaluated using a chi-square test. The Spearman correlation was performed to determine the associations between the variables. A probability value of < 0.05 was considered to indicate statistical significance.

## RESULTS

Ninety-six patients with atherosclerotic cerebral infarction were included into the clinical study, which consisted of 57 males and 39 females, age ranged from 40 to 83 years old. They were divided into the carotid plaque group and non-plaque group according to the existence of a carotid plaque after carotid artery ultrasonography, 78 participants in carotid group and 18 in non-plaque group. The carotid plaque group was further subdivided into stable plaque and unstable plaque subgroups according to plaque characteristics. In 78 participants with plaques, 47 in stable plaque subgroup and 31 in unstable plaque subgroup. There was no significant difference in gender between the stable plaque subgroup, unstable plaque subgroup and non-plaque group (p>0.05), but the age in two subgroups was significantly higher than the non-plaque group (p<0.05), while no significant difference between the two subgroups (p>0.05, Table-I).

**Table-I:** The comparison between stable plaque subgroup, unstable plaque subgroup and non-plaque group.

	Stable plaque subgroup	Unstable plaque subgroup	Non-plaque group	P value
Number	47	31	18	-
Age (year)	68.1±9.2^b^	69.5±10.2^b^	56.7±9.8	P<0.05
Gender(M/F)	27/20	19/12	11/7	p>0.05
hs-CRP(mg/L)	7.18±2.6^ab^	11.7±3.3^b^	2.9±2.1	P<0.05
IMT(mm)	1.19±0.24^ab^	1.37±0.34^b^	0.97±0.09	P<0.05

***Note:*** hs-CRP= high-sensitivity C-reactive protein. IMT= intima–media thickness.

a indicates p<0.05, compared to unstable plaque subgroup.

b indicates p<0.05, compared to non-plaque group.

In terms of IMT and hs-CRP level, the unstable plaque subgroup presented with the highest values, followed by stable plaque subgroup and non-plaque group. There was significant difference in IMT and hs-CRP level between the groups (p<0.05, Table-I).

In terms of the nervous damage, we found with the increase in nervous damage scale, the level of hs-CRP increases significantly (p<0.05), while there was no significant difference in IMT between patients with different nervous damage grades (p>0.05). In addition, there was significant correlation between NIHSS score and hs-CRP in patients with atherosclerotic cerebral infarction (p<0.05) (Table-II).

**Table-II:** The comparison of hs-CRP and IMT in patients with different scale of nervous damage.

Scale	N	hs-CRP(mg/L)	IMT(mm)
Mild	31	4.05±1.05	1.06±0.16
Moderate	39	6.47±2.14	1.18±0.29
Severe	26	11.28±3.19	1.25±0.34
P values		P<0.05	p>0.05

***Note:*** hs-CRP= high-sensitivity C-reactive protein. IMT= intima–media thickness.

## DISCUSSION

In the current study, we tried to detect the correlations of high-sensitivity (hs)-CRP, intima–media thickness, atherosclerotic plaques and neurological function in patients with atherosclerotic cerebral infarction, to facilitate physicians in diagnosing atherosclerotic cerebral infarction.

We found the age of patients in two subgroups was significantly higher than the non-plaque group, this demonstrates that the formation of atherosclerotic plaques may be closely correlated to the age of patients. In a study of 3681 patients using Cox proportional hazards regression analysis, Yang suggested age, sex, and carotid plaque burden can significantly predict risk of ipsilateral stroke or transient ischemic attack, death from stroke, or death from unknown cause at a mean follow-up of 2.56 years.^15^ In the current study, we didn’t analyze the correlation between sex, carotid plaque burden and stroke, but we found the patients in no plaques group were relatively younger, indicating the elderly patients have more risks of stroke, and the viewpoints of the two studies are similar.

Moreover, many studies have confirmed the close correlation between inflammation and atherosclerotic plaque development.^4^,^5^ Both preclinical and clinical investigations demonstrates that inflammatory reactions operates all the stages of atherosclerotic events, and inflammatory process is associated with the risk factors for the formation of atherosclerotic plaque and the modified pathophysiology of the blood vessels.^16^ In terms of IMT and hs-CRP level in the current study, the plaque subgroups presented with the higher values than the no plaques groups. This confirmed the above points, demonstrating the important role of inflammation in atherosclerotic plaque development again. In addition, we found with the nervous damage scale increase, the level of hs-CRP increase significantly, and there was significant correlation between NIHSS score and hs-CRP in patients with atherosclerotic cerebral infarction. This indicates that the increased level of hs-CRP can reflect not only the scale of inflammation, but also the scale of nervous damage. As a result, hs-CRP can be regarded as a high sensitive index in deciding the risk and prognosis of stroke.

### Limitations of the study

First, the sample size was relatively small, a larger scale study may demonstrate the facts more clearly. Second, the values of intima–media thickness came from carotid color ultrasonography, while personal experience and subjective factors may affect the final results of carotid color ultrasonography. But, despite of the limitations, we believe we can make definite conclusions from the present study, facilitating physicians in determining the diagnosis and prognosis of the fatal disease.

## References

[1] Wang B, Sun S, Liu G, Li Y, Pang J, Zhang J (2013). Correlation between aortic/carotid atherosclerotic plaques and cerebral infarction. Exp Ther Med.

[2] Song Y, Liu H, Long L, Zhang N, Liu Y (2015). TLR4 rs1927911, but not TLR2 rs5743708, is associated with atherosclerotic cerebral infarction in the Southern Han population: a case-control study. Medicine (Baltimore).

[3] Jiang B, Wang WZ, Chen H, Hong Z, Yang QD, Wu SP (2006). Incidence and trends of stroke and its subtypes in China: results from three large cities. Stroke.

[4] Luo S, Wang F, Li Z, Deng J (2013). Effect of the +781C/T polymorphism in the interleukin-8 gene on atherosclerotic cerebral infarction, and its interaction with smoking and drinking. PLoS One.

[5] Gregersen I, Holm S, Dahl TB, Halvorsen B, Aukrust P A focus on inflammation as a major risk factor for atherosclerotic cardiovascular diseases. Expert Rev Cardiovasc Ther.

[6] Ridker PM, Hennekens CH, Buring JE, Rifai N (2000). C-reactive protein and other markers of inflammation in the prediction of cardiovascular disease in women. N Engl J Med.

[7] Diener HC, Bogousslavsky J, Brass LM, Cimminiello C, Csiba L, Kaste M (2004). Aspirin and clopidogrel compared with clopidogrel alone after recent ischaemic stroke or transient ischaemic attack in high-risk patients (MATCH): randomised, double-blind, placebo-controlled trial. Lancet.

[8] Spagnoli LG, Mauriello A, Sangiorgi G, Fratoni S, Bonanno E, Schwartz RS (2004). Extracranial thrombotically active carotid plaque as a risk factor for ischemic stroke. JAMA.

[9] Spagnoli LG, Bonanno E, Sangiorgi G, Mauriello A (2007). Role of inflammation in atherosclerosis. J Nucl Med.

[10] Giannoni MF, Vicenzini E, Citone M, Ricciardi MC, Irace L, Laurito A (2009). Contrast carotid ultrasound for the detection of unstable plaques with neoangiogenesis: a pilot study. Eur J Vasc Endovasc Surg.

[11] (1996). The Fourth National Cerebrovascular Diseases, Conference, Diagnostic criteria and disability scale for cerebrovascular, diseases Zhonghua Shenjingke Zazhi.

[12] Rosvall M, Janzon L, Berglund G, Engstrom G, Hedblad B (2005). Incidence of stroke is related to carotid IMT even in the absence of plaque. Atherosclerosis.

[13] Wang YJ, Gong ZQ, Bi XM, Li YL (2015). Correlation of plasma soluble cluster of differentiation 40 ligand, alpha fetoprotein A, and pregnancy-associated plasma protein A with carotid plaque in patients with ischemic stroke. Genet Mol Res.

[14] Wang JH, Zhang YW, Zhang P, Deng BQ, Ding S, Wang ZK (2013). CD40 ligand as a potential biomarker for atherosclerotic instability. Neurol Res.

[15] Yang C, Bogiatzi C, Spence JD (2015). Risk of Stroke at the Time of Carotid Occlusion. JAMA Neurol.

[16] Husain K, Hernandez W, Ansari RA, Ferder L (2015). Inflammation, oxidative stress and renin angiotensin system in atherosclerosis. World J Biol Chem.

